# Implications of *Glutathione Peroxidase 3* Expression in a Cohort of Egyptian Patients with Acute Myeloid Leukemia

**DOI:** 10.31557/APJCP.2020.21.12.3567

**Published:** 2020-12

**Authors:** Yasmine Shaaban, Salah Aref, Mona Taalab, Mohamed Ayed, Mohamed Mabed

**Affiliations:** 1 *Clinical Hematology Unit, Department of Internal Medicine, Faculty of Medicine, Oncology Center, Mansoura University, Mansoura, Egypt. *; 2 *The Hematology Unit, Department of Clinical Pathology, Faculty of Medicine, Mansoura University, Egypt. *

**Keywords:** Acute myeloid leukemia, Glutathione peroxidase 3 (GPX3), hematologic malignancies, prognosis

## Abstract

**Background:**

The impact of low expression of *Glutathione peroxidase 3 (GPX3)* on the clinical course of acute myeloid leukemia (AML) is poorly investigated.

**Aims:**

To explore the status of *GPX3* expression and analyze its clinical characteristics and prognosis in a cohort of Egyptian patients with AML.

**Methods:**

GPX3 mRNA level was assessed by RT-q PCR in 40 newly diagnosed AML patients and 10 healthy controls.

**Results:**

The gene expression level was significantly lower in AML patients than the control group (P < 0.001). A cut off value (0.1223) for the discrimination between AML and controls was obtained by ROC curve. According to this cutoff value; the patients were reassigned into 2 groups; 28 patients with lower *GPX3* expression and 12 patients with high *GPX3* expression. *GPX3*^low^ expression was significantly associated with higher incidence of induction death (P= 0.037) and lower CR rate (P=0.048). Moreover, *GPX3*^low^ expression was significantly associated with shorter cumulative 1-year overall survival (OS) (P = 0.001) and disease-free survival (DFS) (P=0.028).

**Conclusion:**

*GPX3*^low^ expression status is considered a poor prognostic factor in AML predicting shorter OS and DFS. The study highlights the importance of targeting glutathione metabolism as a central component of the anti-leukemia therapy.

## Introduction

Acute myeloid leukemia (AML) is a clonal heterogeneous disorder harboring molecular abnormalities. Mutations acquired by myeloid progenitors result in a proliferative and survival advantage and impair hematopoietic differentiation. Conventional and molecular cytogenetic abnormalities are important factors in patient diagnosis, risk assessment and therapeutic interference. Although many recurrent genetic lesions have been identified, no single mutation is enough to stimulate leukemogenesis. Novel molecular genetic methodologies and genomic sequencing of leukemic cells will help improve the understanding of the pathogenesis of AML and identify novel therapeutic targets (Aref et al., 2014; Aref et al., 2015; Pourrajab et al., 2020).

Glutathione Peroxidase (GPX) is an important enzyme in maintaining the redox balance of the human body. It catalyzes the reduction of hydrogen peroxides (H_2_O_2_) to water; and lipid peroxides to their corresponding alcohols. Eight enzymes of GPX family have been identified in various human tissues. Many studies illustrated that subjects with lower GPX activity are predisposed to impaired antioxidant protection and oxidative damage of their DNA (Ighodaro et al., 2018). GPX3, the most investigated member of GPX family, locates on chromosome 5q23. The down-regulation of GPX3 is usually associated with marked organ damage (Qi et al., 2018). Recent studies postulated that this gene plays a tumor suppressor role in solid neoplasms such as esophageal carcinoma, prostate, thyroid carcinomas, also in head and neck carcinoma (HNC) and other malignancies (Bansal and, Simon, 2018). Studies have been focusing on the role of glutathione peroxidases and other antioxidants in counteracting the oxidative stress and its subsequent effects on DNA damage in various hematological neoplasms as in multiple myeloma (MM) (Mehdi et al., 2018). However, the role of GPX3 in the leukemogenesis of AML is still elusive and recent studies have been trying to correlate between the pattern of *GPX3 mRNA *expression level in newly diagnosed AML patients and its effect on clinical and survival outcomes.

The primary objective of our study was to explore the status of *GPX3 *expression and analyze its clinical characteristics and prognosis in AML.

## Materials and Methods

*Patients*

After obtaining the written informed consent, BM and peripheral blood samples were collected from 40 newly diagnosed AML patients according to the French-America-British (FAB) and the World Health Organization (WHO) criteria [Neame et al 1986]. All patients were treated at the Oncology Center Mansoura University (OCMU), Egypt from January 2017 to December 2018. Treatment protocol was the standard induction chemotherapy 3+7 protocol (3 days Doxorubicin at a dose 30 mg/m^2^ + 7 days Cytrabine at a dose of 200 mg divided every 12 hours). Patients who achieved CR subsequently received consolidation regimen of high dose cytrabine (HiDA) for 3-4 cycles, while refractory cases received salvage therapy. Ten healthy donors were recruited as normal controls.

*GPX3 mRNA level assessment by Real-time quantitative PCR (RT-Q-PCR)*

*RNA extraction step*

RNA was extracted from whole blood samples using Thermo Scientific Gene JET RNA Purification Kit. Twenty μL of 14.3 M β-mercaptoethanol or 2 M DTT were mixed with every 1 mL volume of Lysis Buffer. Blood sample collection and RNA purification were done in the same day. Samples were stored at 4°C. Blood cells were collected by centrifugation of 0.5 mL of whole blood at 400 × g for 5 min at 4°C. A pellet of ~60%-70% of the total sample volume was collected. The clear supernatant from the pellet was removed and resuspend in 600 μL of Lysis Buffer supplemented with β-mercaptoethanol or DTT. 450 μL of ethanol (96-100%) mixed by pipetting and up to 700 μL of lysate were transferred to the GeneJET RNA Purification Column inserted in a collection tube. Centrifugation of the column for 1 min at ≥12,000 × g was done. The flow was discarded and the step was repeated until all of the lysate has been transferred into the column and centrifuged. The GeneJET RNA Purification Column was placed into a new 2 mL collection tube with 700 μL of wash Buffer. 

The purification column was placed back into the collection tube. 600 μL of Wash Buffer 2 in the GeneJET RNA Purification Column were centrifuged for 1 min at ≥12,000 × g.Two hunderds fifty μL of Wash Buffer 2 in the GeneJET RNA Purification Column were centrifuged for 2 min at ≥12,000 × g. The GeneJET RNA Purification Column was then transferred to a sterile 1.5 mL RNase-free microcentrifuge tube. The purified RNA was used for downstream applications or stored at -20°C or -70°C until use.

*Reverse transcriptase step*

This step was done using High-Capacity cDNA Reverse Transcription Kits (Applied Biosystems).

*Preparation of the 2X RT master mix*

The kit components were allowed to thaw on ice. The volume of the components needed was calculated to prepare the required number of reactions. The 2X RT master mix was placed on ice and mixed gently. The reverse transcription reactions were prepared with 10 μL of 2X RT master mixed into each well of a 96-well reaction plate or individual tube. 10 μL of RNA sample were pipetted into each well, pipetting up and down two times to be mixed. The plates or tubes were sealed. The plate or tubes were centrifuged to spin down the contents and to eliminate any air bubbles. The plate or tubes were placed on ice until the time of thermal cycler.

*Real-time PCR step*

For each sample, 2 reaction tubes were prepared (one for the gene *GPX3* and one for the internal control GAPH) *GPX3*: gene expression ready-made assay ID Hs00173566_m1 (catalog #4331182) and *GAPH*: housekeeping gene.

*Statistical analysis*

Statistical analyses were performed using Statistical package for Social Science (IBM Corp. Released 2011. IBM SPSS Statistics for Windows, Version 20.0. Armonk, NY: IBM Corp.). Shapiro test for normality of data distribution was used. Significant data were considered to be nonparametric. Student t Test measured the statistical significance of the difference between the mean of the two study groups. Mann Whitney Test (U test) was used to assess the statistical significance of the difference of a non-parametric variable between the two study groups. Chi-Square and Fisher’s exact tests were used to examine the relationship between two qualitative variables. Correlation analysis was done to assess the strength of association between two quantitative variables. The correlation coefficient defines the strength and direction of the linear relationship between 2 variables. Receiver operating characteristic curve (ROC) and area under the ROC curve (AUC) were con-ducted for estimation of the value of *GPX3* expression in the discrimination of AML patients from healthy controls. Kaplan–Meier test was used for survival analysis and the statistical significance of differences among curves was determined by Log-Rank test. Cox regression analysis of factors potentially related to survival was performed. The P value was considered significant if < 0.05 at confidence interval 95%.

## Results

*GPX3 expression level in controls and AML patients*

Median level of *GPX3* expression in the controls was 0.85 (range; 0.14 -1.53). Median level of GPX3 expression in AML patients was 0.0325 (range; 0.001-1.15), being significantly lower than that of controls (P < 0.001).

*Estimating a value for GPX3 expression*

**Figure 1:**
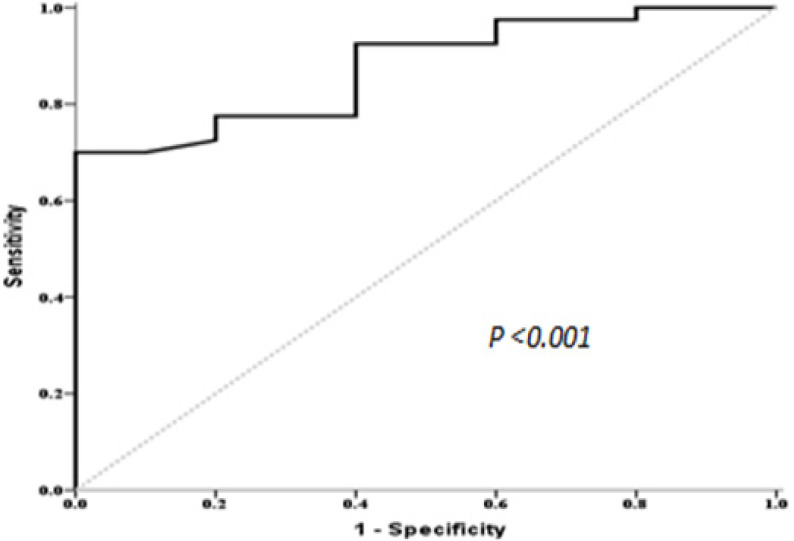
ROC Curve of Serum *GPX3* for Discrimination between AML Patients and Control Group

ROC curve of *GPX3* expression level was performed for discrimination between AML patients and controls. It showed an AUC of 0.876, p <0.001 (Figure 1). At a cut off value of 0.1223, the sensitivity, specificity and accuracy were 70%, 100% and 76%, respectively. GPX3 expression therefore, could serve as a potential biomarker for discriminating between AML and normal controls.

*The relation between GPX3 expression level and the clinical features of AML patients*

The Studied AML patients were reassigned into 2 groups; 28 patients with lower *GPX3* expression (or below the cutoff value) and 12 patients with high *GPX3* expression (or above the cutoff value). There were no significant differences in age or gender distribution (P=0.793 and 0.836). Also, there were no significant differences in CBC parameters, peripheral blood or BM blasts between the two groups (P > 0.05). No significant differences in the distribution of FAB classification or the cytogenetic data between the 2 groups (P > 0.05). The frequency of low *GPX3* expression in favorable and poor karyotypes were similar (10.7%, 3/28), while its frequency in intermediate risk group was higher (78.6%, 22/28) with no significant difference as regard risk classification between the 2 groups (P = 0.804). In this study; decreased *GPX3* expression level in AML was associated with poor outcome, it had higher frequency in intermediate/poor karyotyping categories when compared to the other group (Table 1). 

*The association between GPX3 expression and the clinical outcome*

**Figure 2:**
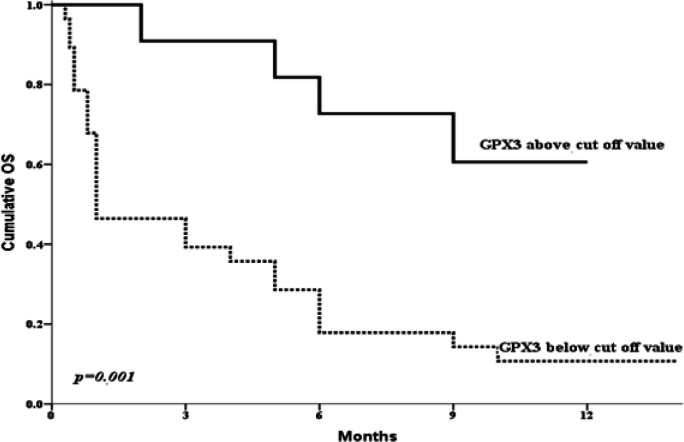
OS According to *GPX3* Cut off Value of Obtained by ROC Curve in All Studied AML Patients

**Figure 3:**
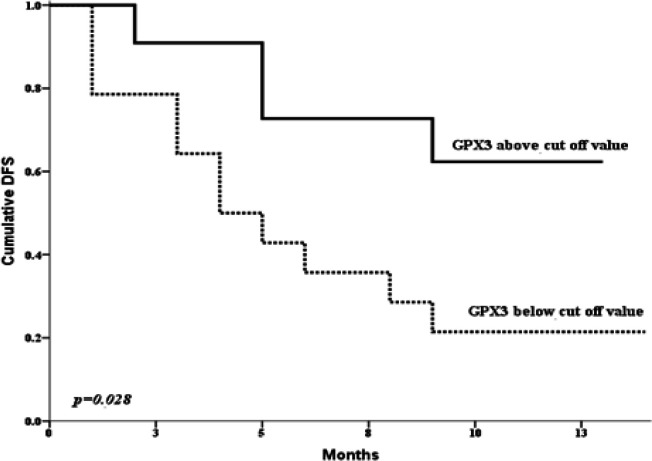
DFS According to *GPX3* Cut off Value of Obtained by ROC Curve in All Studied AML Patient

The association between the expression of GPX3 and the prognosis in AML patients conducted by Cox regression analysis showed that lower *GPX3* expression group was significantly associated with lower complete remission (CR) (P = 0.048) and higher mortality rates (P = 0.001). Our study demonstrated that decreased *GPX3* expression level was significantly associated with shorter cumulative 1-year OS and DFS compared to ‘’high* GPX3 *expression’’ subgroup [median 1 month vs. 9.5 months for OS, P = 0.001 and 4 months vs. 10 months for DFS, P = 0.028, respectively (Figures 2 and 3). Moreover, the uni and multivariate analysis disclosed the prognostic impact of low *GPX3* expression as a poor prognostic marker for prediction of shorter OS within AML patients (Table 2).

## Discussion

*Glutathione peroxidase 3 (GPX3) *belongs to GPX family, its main function as an antioxidant is to get rid of harmful free radicals (FR) and detoxify toxins from cells. It is implicated in cellular proliferation, division, and differentiation (Bansal and Simon, 2018). *GPX* genes may have different frequencies in various human tissues but they all seem to have high similarity in their composition and antioxidant functions. It has been postulated that this enzyme has a pivotal role in regulation of apoptotic genes like *Bax *and *Bcl-2* (Chang et al., 2018).

Different studies suggested that *GPX3* expression may have an impact on the pathogenesis of various tumors and that it serves as a tumor suppressor gene and its deficiency interferes with tumorogenesis. Furthermore, studies have been trying to detect the effect of lower *GPX3 mRNA* levels on clinical and survival outcomes. However, its role in hematological neoplasms remains under evaluated. Herault and colleagues found that this gene is crucial for the competitiveness of leukemia stem cells (LSCs) and the self-renewal of hematopoietic stem cells (HSCs) and *GPX3* levels were correlated with the self-renewal activity of LSCs (Herault et al., 2012).

Another study detected a method for enhancing target therapy in AML by eradication of primitive CD34+ LSCs that have acquired aberrant glutathione metabolism using agents that target aberrant glutathione pathway such as parthenolide (PTL) or piperlongumine and/or piperlongumine (PLM) together with cytarabine and idarubicin on AML specimens. These agents induced almost complete glutathione depletion and severe cell death in CD34+ AML cells and proposed that these possible novel agents could overcome chemotherapy resistance in comparison to standard chemotherapy agents (Pei et al., 2013).

Zhou et al., (2015) showed that down-regulation of the expression of GPX3 was significantly associated with favorable/intermediate cytogenetics. However, they failed to show an impact of GPX3 low expression level on clinical response or survival in the studied AML patients. 

Other studies concluded that GPX3 methylation which is an epigenetic mechanism that affects *GPX3 *expression levels, was not significantly associated with differences among karyotype classification but was significantly associated with poor molecular genomic. GPX3 methylation was increased in patients with myelodysplastic syndrome (MDS) progressing or transforming to secondary AML (sAML). GPX3 was postulated to be an independent biomarker to discriminate between AML and normal controls and it acts as an adverse biomarker (Qi et al., 2015; Zhou et al., 2015).

We herein, explore the *GPX3 *expression status and analyze its impact on clinical characteristics and outcome in forty newly diagnosed AML patients. We confirmed its role in the prognosis of these patients. Our study revealed that the level of *GPX3 *expression was lowered in newly diagnosed AML patients. Moreover, the ROC curve analysis disclosed that *GPX3* expression could be a promising biomarker for discriminating AML from normal subjects.

Our data of the frequency of *GPX3* expression status in different cytogenetic and karyotyping classifications demonstrated that low *GPX3* expression was insignificantly equal in patients with favorable/poor karyotypes. This did not coincide with a previous study where the increased level of *GPX3* expression was more frequent in high risk karyotypes; MLL rearrangement other than t(9;11), -7 and complex karyotype (≥ 3 chromosomal abnormalities) (Zhou et al., 2017).

The association between *GPX3* expression and its promoter methylation has been found in different malignancies (Falck et al., 2010). Hypermethylation of GPX3 in multiple myeloma (MM) may be involved in drug response and interaction with the BM microenvironment. MM patients treated with bortezomib have a higher global DNA methylation, which is associated with a higher OS than patients with a low global DNA methylation (Issa et al., 2017). 

The BM microenvironment plays a crucial role in the regulation of the oxidative metabolism of leukemic cells by promoting the inhibition of the H2O2/p38 MAPK axis via the induction of GPx3. Modulation of the GPx3/H_2_O_2_/p38 MAPK pathway could be a extended in clinical trials for leukemia patients for finding novel methods in targeting LSCs in the microenviroment (Zhang et al., 2018).

GPX3, an antioxidant reactive oxygen species (ROS) scavenger, so its downregulation leads to ROS activation and activation of oncogenic signaling pathways such as Ras, c-Myc, or BCR-ABLl; which contribute in further increase of ROS levels; which in turn affects both nuclear and mitochondrial DNA by promoting genomic instability (Kim et al., 2016). FLT3-ITD induces ROS production through NADPH oxidases which has been suggested to contribute to leukaemic transformation (Jayavelu et al., 2016). The 2-HG oncometabolite found in IDH1 and IDH2 mutations in AML patients, facilitates leukaemic transformation by increasing ROS, and thus IDH1 and 2 inhibitors can be seen as a promising strategy against AML and they can counteract the ROS effects which increase in patients with low expression levels of GPX3 (Fujii et al., 2016). BCL-2 inhibition was shown to disrupt mitochondrial energy production, which increased ROS and induced apoptosis in LSCs, with little or no impact on normal stem cells (Bigarella et al., 2014). Venetoclax, a BCL-2 inhibitor, -based therapies allowed for rapid responses and were able to effectively target the LSCs population (Pollyea et al., 2019).

Zhuo et al., (2015) showed that GPX3 mRNA level was significantly increased and GPX3 promoter methylation level was decreased after treated THP1 cell line by 5 azacytidine 2 deoxycytidine [5-aza-dC]. From this study and others, GPX3 expression could be up-regulated after 5-aza-dC treatment in different cancer cell lines.

GPX activity was significantly low in a previous study on adult AML Egyptian patients versus control subjects (P value <0.01). GPX activity significantly increased in AML patients after treatment. Decreased selenium level and reduced GPX activity in AML patients supported the association between carcinogenesis and subnormal Se states (Asfour et al., 2009). 

Taken together, our results indicate that low GPX3 expression level is considered a poor prognostic factor in AML predicting shorter OS and DFS. Further studies are needed to explore the GPX3 promoter methylation pattern in AML patients and to understand the role of GPX3 as tumor suppressor gene and its relation with molecular cytogenetics involved in AML. Also, it highlights the importance of targeting glutathione metabolism as a central component of the anti-leukemia therapy.

**Table 1:** Association between *GPX3* Expression Level and Clinical Parameters in Newly Diagnosed AML Patients

Patient's parameters	Status of serum *GPX3* expression level *GPX3* below cut off value ''Low* GPX3* expression" (No=28)	*GPX3 *above cut off value "High *GPX3* expression" (No=12)	P value
Gender, male/female	13/15	6/6	0.836
Median age, years	43.9	45.3	0.793
Median TLC, x 10^9^/L (range)	31.5 (1.9-300)	34.9 (1.7-150)	0.813
Median Hb, x 10^9^/L (range)	8.5 (2.8-12.6)	8.2 (6.5-14.2)	0.941
Median Plt, x 10^9^/L (range)	32.2 (16-170)	35.2 (7-320)	0.965
Median ANC, x 10^9^/L (range)	1.4 (0-37.9)	2.6 (0-90.5)	0.941
FAB 0.064			
M0	0 (0%)	0 (0%)	
M1	7 (25%)	1 (8.3%)	
M2	6 (21.4%)	4 (33.3%)	
M4	6 (21.4%)	7 (58.3%)	
M5	7 (25%)	0 (0%)	
M6	2 (7.1%)	0 (0%)	
M7	0 (0%)	0 (0%)	
Cytogenetics > 0.05			
t(8;21)	1(3.6%)	0 (0%)	
inv (16)	3 (10.7%)	1 (8.3%)	
t(6;9)	1 (3.6%)	0 (0%)	
11q23	1 (3.6%)	0 (0%)	
-7	1 (3.6%)	0 (0%)	
8	0 (0%)	1 (8.3%)	
NK	21 (75%)	10 (83.3%)	
Others	1 (3.6%)	0 (0%)	
Karyotyping classification or Risk Assessment 0.804			
Favorable	3 (10.7%)	1 (8.3%)	
Intermediate	22 (78.6%)	11 (91.7%)	
Poor	3 (10.7%)	0 (0%)	
Complete Remission	14 (50%)	10 (83.3%)	0.091

TLC, Total leucocytic count; Hb, hemoglobin; ANC, Absolute Neutrophile Count; Plt, platelets; PB, Peripheral blood; BM, Bone marrow

**Table 2:** Cox Regression Analysis for Prediction of OS within the Studied AML Patients

	Univariate				Multivariate			
	p	HR	95% CI		p	HR	95% CI	
Age	0.92	1.001	0.976	1.027				
Gender	0.577	1.234	0.59	2.578				
ANC (X10^9^/L)	0.928	0.999	0.981	1.018				
Peripheral blasts (%)	0.993	1	0.985	1.015				
BM Blast (%)	0.28	1.01	0.992	1.029				
LDH	0.018	1.012	1.005	1.071	0.031	1.023	1.002	1.091
Normal karyotype	0.784	0.888	0.378	2.082				
*GPX3* expression	0.024	0.106	0.015	0.747	0.033	0.113	0.015	0.844
